# Progress in Ophthalmology

**Published:** 1901-12-14

**Authors:** 


### PROGRESS IN OPHTHALMOLOGY.

(*Continued from page* 169.)

**Strabismus.**[3]—In a clinical lecture delivered by Priestly Smith on the subject of internal strabismus at the Medical Graduates' College and Polyclinic, the subject is treated exhaustively. He says: "It is very desirable that every medical man should have a clear idea as to the nature of the disorder and the kind of treatment which is required." The special point, he urges, is that at the present time harm is done in many of these cases by delay of treatment. The advice is too often given, "Do nothing at present; wait a few years and then, if necessary, have an operation." This is usually bad advice. He goes on further to say the nature of the disorder is this: there is no paralysis and no fault of the muscle, but the convergent centre acts more than it should, and the conjugate centre rectifies the deviation of one eye and doubles that of the other. If the child has two equally good eyes, he will sometimes use the right and squint with the left and vice versâ. If one eye is better than the other, he will generally use the better, and squint with the worse. He attributes the over action of the convergence centre, to an effort to overcome a hypermetropia—in 90 cases out of 100 the child has hypermetropia and therefore requires glasses. If the 90 cases are put into proper glasses and examined a week later, he maintains, that nearly all will be better; that the squint is less pronounced than before; and that in a considerable number of cases it is actually gone—proving that the hypermetropia was, to some extent, at least, the cause of the squint; therefore if a child squints, and is not in spectacles, the strong probability is that he ought to be. The explanation given is this. The hypermetrope, whether he looks at a distant or a near object, must make a greater effort of accommodation than should be the case. Accommodation is associated with convergence. The two efforts are always made together; the centres which preside over them lie close together in the brain; it is difficult to make the one effort without the other. Hence the children who accommodate to excess converge to excess. Why do not all hypermetropes squint? As a rule a hypermetropic child learns to control these efforts in the way which is necessary for his peculiar condition; he learns to accommodate to excess while he converges normally. Those who cannot learn and maintain this habit have to squint. A large number of cases of squint come out during an attack of whooping-cough, or during or after measles, or scarlatina, or in connection with convulsions, or a slight accident or something which upset the child's nervous balance. As an example of the last cause, he quotes a case of a child who went to school and got on well for a year and was never known to squint. One day, for some fault, she was put in a corner with a cloth over her head, was ashamed and frightened, and came home with a bad squint. Any sudden shock to the nervous centre may bring on a squint; anything which upsets the nervous balance paralyses the higher mental control. Bandaging one eye for two or three weeks, if the patient happens to be hypermetropic is apt to cause a squint to develope. While the eye is covered there is no inducement to keep it straight. Having tabulated a large number of squint cases, he finds that more cases begin between three and four years of age than at any other period—a time when the visual apparatus is not yet fully developed, when its functions are not firmly established, and therefore are easily upset. In a group of children, all of whom began to squint before two years of age, and all of whom had been untreated for three years, 87 per cent. had lost the power of fixation with the squinting eye, whereas in children who began squinting later, and were treated sooner, the proportion was much smaller, until among those who did not begin to squint until they were five years old, no loss of fixation power was found. As to the cure, if the fixation power is lost, it is sure to be a difficult case—the first step in the treatment is almost always to order glasses, which will correct the hypermetropia. If an insignificant amount, it is not necessary, but if +2D or more, then glasses must be procured. The glasses must fit. A common reason why glasses do not succeed is that they fit badly, they rest too low on the face, and the child looks over them; he states that even children under two years of age, who require spectacles, can be taught with little difficulty to wear them. The second step, which is not much less important but much less practised, is the putting of a pad or shade over the good eye in order to make the child use the squinting eye. The best plan is to put a pad of Gamgee tissue between the closed eye and the spectacles. At first the child will pull it off 20 times a day, and the mother will have to replace it

21 times; but in a few days the child gets accustomed to it and will learn in that way to use the squinting eye. The value of this treatment is beyond question. Even eyes which have no power fixation at first will sometimes recover it after a few weeks in this way. At to operation, the rule should be to cure the squint without operation, if possible, to watch the progress systematically, and so long as any improvement goes on to do nothing more; but when progress by these means comes definitely to a standstill, and an operation offers the only prospect of recovering binocular vision, then to operate, at once, whatever the age of the child.

[5] Clinical Jour., Sept. 18, 1891, Vol. XVIII., No. 22.

